# Immunological Monitoring of Renal Transplant Recipients to Predict Acute Allograft Rejection Following the Discontinuation of Tacrolimus

**DOI:** 10.1371/journal.pone.0002711

**Published:** 2008-07-16

**Authors:** Ellen Kreijveld, Hans J. P. M. Koenen, Bram van Cranenbroek, Esther van Rijssen, Irma Joosten, Luuk B. Hilbrands

**Affiliations:** 1 Department of Blood Transfusion and Transplantation Immunology, Radboud University Nijmegen Medical Centre, Nijmegen, the Netherlands; 2 Department of Nephrology, Radboud University Nijmegen Medical Centre, Nijmegen, the Netherlands; L' Istituto di Biomedicina ed Immunologia Molecolare, Consiglio Nazionale delle Ricerche, Italy

## Abstract

**Background:**

Transplant patients would benefit from reduction of immunosuppression providing that graft rejection is prevented. We have evaluated a number of immunological markers in blood of patients in whom tacrolimus was withdrawn after renal transplantation. The alloreactive precursor frequency of CD4+ and CD8+ T cells, the frequency of T cell subsets and the functional capacity of CD4+CD25+FoxP3+ regulatory T cells (Treg) were analyzed before transplantation and before tacrolimus reduction. In a case-control design, the results were compared between patients with (n = 15) and without (n = 28) acute rejection after tacrolimus withdrawal.

**Principal Findings:**

Prior to tacrolimus reduction, the ratio between memory CD8+ T cells and Treg was higher in rejectors compared to non-rejectors. Rejectors also had a higher ratio between memory CD4+ T cells and Treg, and ratios <20 were only observed in non-rejectors. Between the time of transplantation and the start of tacrolimus withdrawal, an increase in naive T cell frequencies and a reciprocal decrease of effector T cell percentages was observed in rejectors. The proportion of Treg within the CD4+ T cells decreased after transplantation, but anti-donor regulatory capacity of Treg remained unaltered in rejectors and non-rejectors.

**Conclusions:**

Immunological monitoring revealed an association between acute rejection following the withdrawal of tacrolimus and 1) the ratio of memory T cells and Treg prior to the start of tacrolimus reduction, and 2) changes in the distribution of naive, effector and memory T cells over time. Combination of these two biomarkers allowed highly specific identification of patients in whom immunosuppression could be safely reduced.

## Introduction

Currently, 1-year graft survival rates after renal transplantation are exceeding 90%. However, the life-long administration of immunosuppressive drugs is accompanied by many side effects. Next to increased risk of infections and malignancies, the use of the calcineurin inhibitors (CNI) cyclosporine and tacrolimus is associated with nephrotoxicity, which can contribute to long-term graft failure [1], [2]. Withdrawal of CNI, once stable graft function is achieved, has therefore been attempted in several studies but was associated with an increased risk for acute rejection [3], [4]. On the other hand, successful discontinuation of CNI results in improved renal function and blood pressure, and long-term follow up of patients after CNI withdrawal has shown a favorable outcome [5], [6]. Thus, it is highly desirable to identify transplant patients in whom CNI withdrawal can be successful. To this end, there is a need for in vitro monitoring tools.

CD4+ and CD8+ effector T cells play a central role in the pathogenesis of allograft rejection [7]. CD4+CD25+FoxP3+ regulatory T cells (Treg) are involved in maintaining tolerance towards self antigens, and they can regulate alloreactivity as well [8], [9]. The quantification of alloreactivity, in terms of balance between effector cells and Treg, may allow the identification of patients at risk for acute rejection. Early attempts to characterize alloreactivity have focused on the functional capacity of alloreactive T cells in mixed lymphocyte reactions (MLR), but these assays showed little predictive value in transplantation [10]. Better information was provided by MLR-based limiting dilution assays, estimating the precursor frequencies of cytotoxic T lymphocytes (CTLp) and helper T cells (HTLp) [11]. In bone marrow transplantation, high CTLp frequencies were associated with prolonged leukemia free survival time [12]. However, the benefit of CTLp assessment in solid organ transplantation remains controversial [13], [14]. Using Elispot assays, low numbers of donor-specific IFN-γ producing T cells were associated with stable long-term renal function [15], [16]. Moreover, a high pre-transplant reactivity to a panel of allogeneic stimulator cells was correlated with an increased risk for acute rejection after renal transplantation [17]. Protein profiling of serum to predict rejection has shown promising results in experimental models, but these data have to be confirmed in clinical studies [18].

To date, studies on the role of Treg in alloreactivity are limited due to the low number of Treg in the circulation. The indirect assessment of Treg function in depletion assays, showed that Treg are able to regulate anti-donor responses after transplantation [19], [20], but firm evidence for their role in protection against rejection remains to be provided.

In this study, the level of immunosuppression was reduced to a CNI free regimen in renal transplant patients with stable graft function, according to a standard protocol. Blood samples were collected before transplantation and before the start of tacrolimus withdrawal. Using a case-control design, we compared the T cell subset distribution and *ex vivo* T cell responses between patients who experienced an acute rejection period following the reduction of immunosuppression and patients in whom immunosuppression was reduced successfully.

## Methods

### Transplant patients and immunosuppression

Patients received a renal allograft in our hospital between January 2003 and December 2004. Immunosuppression consisted of tacrolimus in combination with mycophenolate mofetil (MMF) and prednisolone. Patients received 100 mg of prednisolone intravenously during the first 3 days after transplantation and subsequently an oral dose of 15–25 mg/day, tapered to a maintenance dose of 0.1 mg/kg/day. Tacrolimus was started at day 1 or 2 after transplantation at 0.15 mg/kg/day and the dose was subsequently adjusted to achieve whole-blood trough concentrations of 15–20 ng/mL during days 0–14, 10–15 ng/mL during weeks 3–6, and 5–10 ng/mL from week 7. Whole blood tacrolimus concentrations were measured by the IMx analyzer (Abbott Laboratories, Abbott Park, IL, USA). MMF was administered at 1000 mg twice daily with a dose reduction to 750 mg twice daily at 2 weeks after transplantation. Induction therapy with polyclonal or monoclonal antibodies was not used.

At 4 months after transplantation, patients were selected for reduction of their immunosuppression (including withdrawal of tacrolimus) when they met the following inclusion criteria: stable graft function, and at least 1 HLA-B and 1 HLA-DR match between donor and recipient. Patients who received a kidney from a HLA-identical living donor, patients with two or more previously failed grafts, patients with PRA >85%, non-Caucasian patients, and patients that had experienced a steroid-resistant acute rejection episode after their current transplantation were excluded. In addition, patients with severe osteoporosis and patients with bone marrow depression were not included. At first instance, MMF was substituted for azathioprine (Aza, 3 mg/kg daily). The dose of Aza was adjusted in case of leukocytopenia or elevated liver enzymes. When patients did not tolerate a minimum Aza dose of 2 mg/kg/day, MMF was reintroduced. Two months later, six months after transplantation, the tacrolimus dose was gradually reduced to zero over a period of 4 weeks. Meanwhile, the prednisolone dose was increased to 0.15 mg/kg/day. The resulting immunosuppressive therapy after conversion consisted of azathioprine (at least 2 mg/kg/day; otherwise MMF 750 mg twice daily) and prednisolone (0.15 mg/kg/day). All patients were evaluated for acute rejection episodes during the first 6 months after withdrawal of tacrolimus. When there was a deterioration of graft function without clear prerenal or postrenal cause, a graft biopsy was taken. Protocol biopsies at fixed time points were not performed. The study was approved by the Intstitutional Review Board of the Radboud University Nijmegen Medical Centre. All participants gave written informed consent.

### Cell isolation and culture conditions

Blood samples (20 ml) were collected before transplantation (T0) and prior to tacrolimus withdrawal (T1). Donor cells were obtained from peripheral blood (living donors) or spleen tissue (deceased donors). For 3^rd^ party controls, buffy coats were obtained from healthy blood donors (Sanquin Blood Bank region South East, Nijmegen, the Netherlands). Cell isolation and culture were conducted as described elsewhere [21]. All cells from donors and recipients were frozen prior to use in analyses. HLA typing was conducted according to ASHI standards.

### Expression of cell surface markers and of FoxP3

PBMC (1*10^5^) were labeled with fluorochrome-conjugated monoclonal antibodies in PBS-BSA buffer for 20′ at room temperature (RT) in the dark. Samples were measured on a Coulter Epics XL flowcytometer (Beckman Coulter, Fullerton, CA) and analyzed using Coulter Epics Expo 32 software. The following antibodies were used: anti-CD8-FITC (DK25), anti-CD27-FITC (M-T271), anti-CD45RO-FITC (UCHL1), anti-CD25-PE (M-A251), anti-CD28-PE (CD28.1), anti-CD45RA-PE (HI100), anti-CD3-ECD (UCHT1), anti-CD62L-ECD (DREG56), anti-CD8-PC5 (B9.11), and anti-CD4-PC5 (13B8.2). FoxP3-FITC (PCH101) staining was performed according to the manufacturer's procedures (eBioscience, San Diego, CA, USA). Appropriate isotype control mAbs were used for marker settings.

### CFSE-based MLR

The number of donor- and 3^rd^ party-reactive CD4+ and CD8+ T cells was determined using a CFSE-based mixed lymphocyte reaction (CFSE-MLR) [22], [23]. Patient PBMC (10*10^6^) were labeled with 1 µM CFSE (Molecular Probe, Eugene, OR, USA) in CFSE labeling buffer (PBS containing 0.02% HPS) for 10 minutes at RT in the dark and the labeled cells (1*10^5^) were stimulated with irradiated (30 Gy) PKH (Sigma-Aldrich, St Louis, USA) labeled donor cells (1*10^5^) in 96-wells round bottom plates. As controls, patient PBMC were cultured with either PKH labeled irradiated pooled 3^rd^ party PBMC, cultured alone (negative control), or cultured in the presence of anti-CD3/antiCD28 expander beads (positive control). At day 6, the cells were stained for CD4 and CD8.

### Expansion and functional analysis of CD4+CD25^high^ regulatory T cells

Patient PBMC (5–10*10^6^) were incubated with CD4-FITC (clone MT310, Dako, Glostrup, Denmark) and CD25-PE (clone M-A251, BD Bioscience, USA) for 20 minutes at RT in the dark. CD4^+^CD25^high^ and CD4^+^CD25^neg^ control cells were separated using high purity fluorescence activated cell sorting (FACS, Altra flowcytometer, Beckman Coulter, USA). Sorted CD4^+^CD25^high^ and CD4^+^CD25^neg^ T cells were expanded as described previously [21]. To analyze their suppressive potential, a primary MLC was set up, consisting of 4^th^ party responder PBMC and donor or 3^rd^ party stimulator cells. Fourth party responder PBMC were used instead of patient PBMC because of the low proliferative response of the patient PBMC to donor stimulator cells (likely due to close HLA matching), which hampered the read-out of the putative suppression by Treg. The expanded CD4+CD25^high^ and CD4+CD25^neg^ (control) cells were added at increasing ratios to this MLC. It was expected that in the MLC with donor stimulator cells (and not 3^rd^ party stimulator cells), the added donor-specific Treg would be activated and inhibit the proliferation of the 4^th^ party responders by means of linked suppression. Proliferation was measured by ^3^H incorporation.

### Statistical analysis

Patients who experienced an acute rejection after tacrolimus withdrawal and a control group of non-rejecting patients, were as closely as possible matched for the following items: age of donor and recipient (<50 versus ≥50), total number of HLA-mismatches, percentage PRA (<5%, ≥5%), and first or re-transplantation. Data obtained from flow cytometry and CFSE-MLR analyses were compared between the two groups using a Mann-Whitney U-test. For analysis of changes in time within patients a Wilcoxon signed rank test was used. Correlation between values of different time points was analyzed with Spearman's rank test. A p-value ≤0.05 was considered statistically significant.

## Results

### Patient characteristics

Sixty-six patients fulfilled the inclusion criteria and were treated according to the protocol. Twenty-four patients (36%) experienced an acute rejection following the withdrawal of tacrolimus. In 22/24 patients the rejections were confirmed by histology according to the 2001 revised Banff classification [24]. In two patients, the clinical picture and response to therapy was compatible with acute rejection, but no graft biopsy was performed for logistical reasons. All acute rejection episodes were treatable and none of the patients lost their graft within the follow-up period of six months after tacrolimus withdrawal.

In 15 cases with acute rejection sufficient material from donors as well as recipients was available to perform the analyses described below. For each case, we selected two controls from the group of non-rejectors (n = 42), based on optimal matching for a set of relevant parameters (as described in materials and methods section). Because sufficient material was not available for all donors and recipients, the ultimate number of controls was 28. Except for a difference in the degree of HLA-DR matching, the clinical characteristics did not differ significantly between rejectors and non-rejectors (Table 1).

**Table 1:** Patient characteristics.

	Rejectors (n = 15)	Non-rejectors (n = 28)	p-value
Gender (male/female)	9/6	18/10	0.78
Patient age (years)	43±15	40±13	0.65
Donor age (years)	49±14	49±10	0.89
Donor living/deceased	6/9	8/20	0.45
HLA mismatches (0/≥1)			
HLA-A	5/10	15/13	0.21
HLA-B	4/11	9/19	0.71
HLA-DR	0/15	8/20	0.02
Cold ischemia time (hours)*	20±8	17±5	0.30
PRA pretransplant (≥5%)	1	6	0.19
Retransplantation	0	6	0.05
Acute rejection prior to inclusion	1	0	0.17
Azathioprine/MMF at start of tacrolimus withdrawal	11/4	19/9	0.71

* deceased donors only

### Frequencies of effector, memory and regulatory T cell subsets

**Figure 1:**
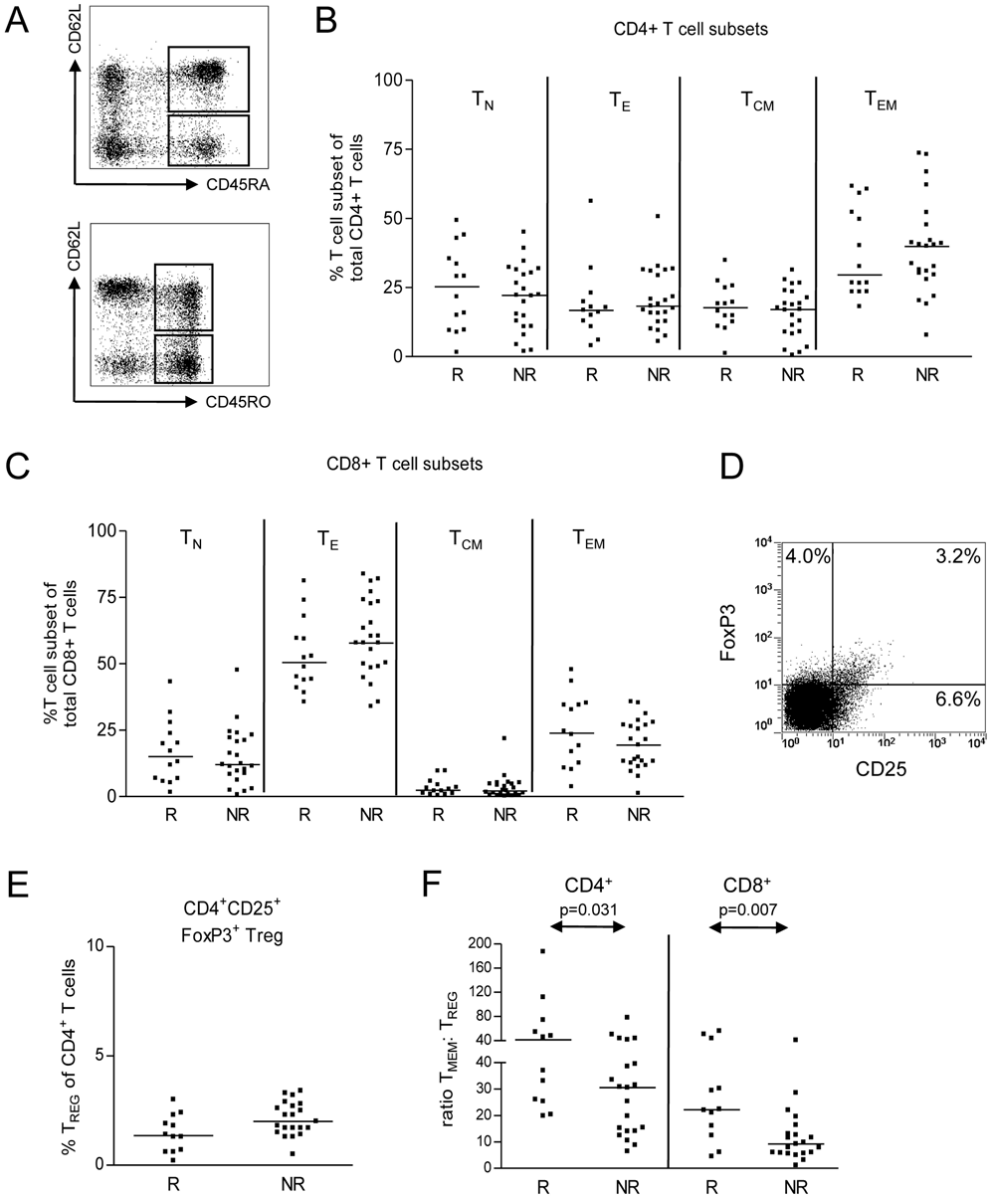
The frequency of effector, memory and regulatory T cell subsets in the peripheral blood of renal transplant patients immediately before the start of tacrolimus dose reduction (T1). (A) Representative dot plots of CD62L versus CD45RA and CD62L versus CD45RO. The analysis was performed on cells within the lymphocyte gate in the forward/side scatter plot. (B) The percentage of naïve (T_N_, CD45RA+CD62L+), effector (T_E_, CD45RA+CD62L-), central memory (T_CM_, CD45RO+CD62L+) and effector memory (T_EM_, CD45RO+CD62L-) T cells within the total CD4+ T cell subset. R vs. NR = not significant. (C) As described under A, for CD8+ T cells. R vs. NR = not significant. (D) Representative dot plot of CD25 versus FoxP3 in CD4+ lymphocytes. (E) The percentage of CD25+FoxP3+ regulatory T cells (Treg) within CD4+ lymphocytes. R vs. NR = not significant. (F) The ratio between the percentage of memory T cells and the percentage of Treg.

T cell subset distribution was analyzed in blood samples collected immediately before the start of tacrolimus dose reduction (T1). Using antibodies directed against CD62L and isoforms of CD45, naive (T_N_, CD45RA+CD62L+), effector (T_E_, CD45RA+CD62L-), effector memory (T_EM_, CD45RO+CD62L-) and central memory (T_CM_, CD45RO+CD62L+) T cell subsets were identified [25], [26]. Neither in the CD4+ T cell pool, nor in the CD8+ T cell compartment did the distribution of the four subsets differ between rejectors and non-rejectors (Figure 1). A similar discrimination between naïve, effector and memory T cells was made on the basis of CD27 and CD28 expression which neither revealed significant differences between rejectors and non-rejectors (data not shown).

Immediately prior to the start of tacrolimus dose reduction, the median frequency of Treg was 1.6% and 2.0% of the total CD4+ T cell subset for rejectors and non-rejectors, respectively (NS; Figure 1E).

Next to the size of the T cell subsets, the balance between reactive and regulatory T cells might be an important determinant of the risk of rejection [27]–[29]. Interestingly, the ratio between the percentage of CD8+ memory T cells (T_EM_ and T_CM_ combined) and the percentage of Treg was significantly higher in rejectors compared with non-rejectors (p = 0.007). A similar difference was observed in the CD4+ T cell compartment (p = 0.032), where low ratios (<20) were only observed for non-rejectors (Figure 1F). Since the group of non-rejectors contained 8 patients without DR mismatches, while none of the rejectors were fully DR matched, we repeated the analysis after the exclusion of DR matched transplantations. Again, the ratio between CD8+ memory T cells and Treg as well as the ratio between CD4+ memory T cells and Treg were higher in rejectors (p = 0.004 and p = 0.01, respectively). To get informed about the reproducibility of this parameter, we also measured the ratio between memory T cells and Treg in blood samples taken two months prior to the start of tacrolimus withdrawal, and compared these values with those obtained immediately before the tacrolimus dose reduction. For the ratio between CD8+ memory T cells and Treg the values did not differ significantly between both time points and the correlation coefficient was 0.89 (P<0.001). For the ratio between CD4+ memory T cells and Treg the values of both time points did not differ either, and the correlation coefficient was 0.74 (P<0.001). We therefore conclude that the variability in time of this parameter is limited.

### CD4+ and CD8+ alloreactive T cell precursor frequency and mitotic activity

**Figure 2:**
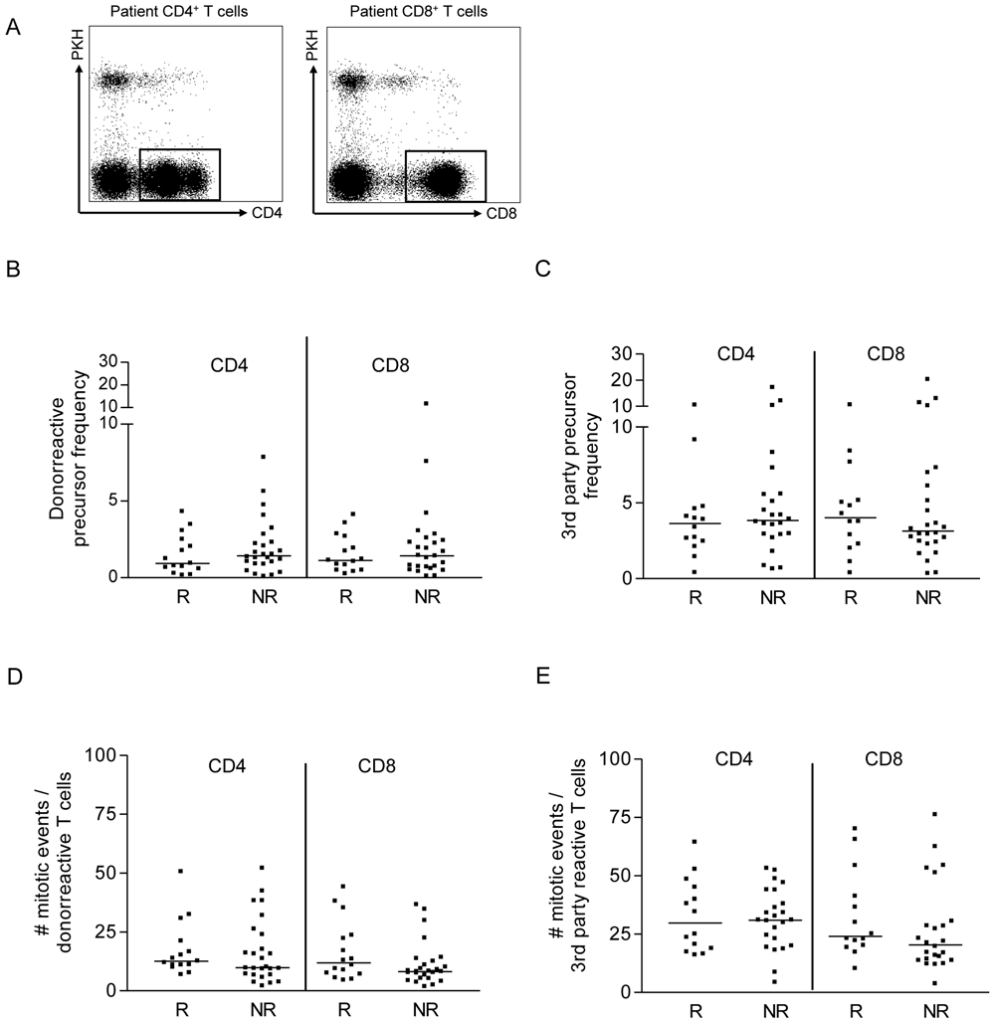
The functional capacity of allo- and 3^rd^ party-reactive CD4+ and CD8+ T cells and CD4+CD25^high^ regulatory T cells isolated from the peripheral blood of transplant patients immediately before the start of tacrolimus dose reduction (T1). A CFSE-MLR was used to determine the precursor frequency and mitotic events of alloreactive CD4+ (left) and CD8+ (right) T cells. CFSE labeled patient PBMC (1*10^5^) were stimulated with PKH labeled donor or 3^rd^ party cells (1*10^5^) for 6 days. Using flow cytometry, patient T cells were gated based on forward/side scatter and PKH exclusion and subsequent CD4 and CD8 staining. An example is shown in (A). The precursor frequency and mitotic events of alloreactive CD4+ and CD8+ T cells were calculated on the basis of the CFSE dilution pattern using Modfit LT™ software [22]. (B) The precursor frequency of donor reactive CD4+ and CD8+ T cells. (C) The precursor frequency of 3^rd^ party reactive CD4+ and CD8+ T cells. (D) The number of mitotic events of alloreactive CD4+ and CD8+ T cells. (E) The number of mitotic events of 3^rd^ party reactive T cells. R vs. NR = not significant.

The precursor frequencies of donor reactive CD4+ and CD8+ T cells ranged from 0.1–6.8%, increasing with the number of HLA mismatches, and did not differ between rejectors and non-rejectors (Figure 2B). Differences were neither observed in the precursor frequencies of 3^rd^ party-reactive CD4+ or CD8+ T cells (Figure 2C).

The number of mitotic events was higher in third party alloreactive T cells as compared to donor reactive CD4+ and CD8+ T cells, but did not differ between rejectors and non-rejectors (Figures 2D and 2E).

### Suppressive potential of Treg

**Figure 3:**
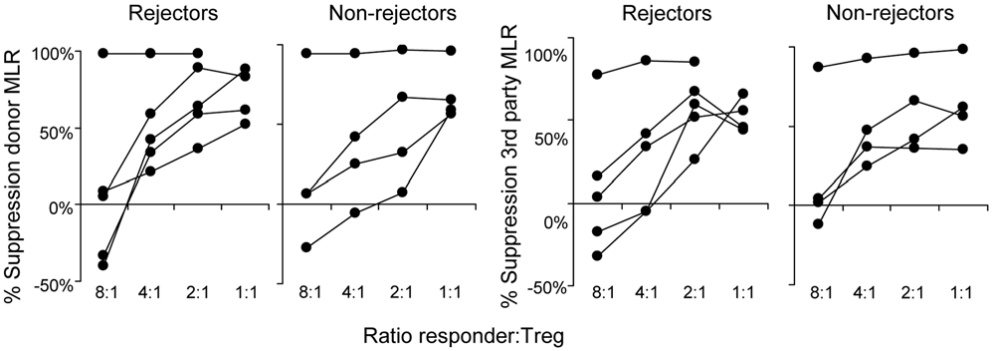
The suppressive potential of Treg isolated from transplant patients immediately before the start of tacrolimus dose reduction. R = rejectors (n = 5), NR = non-rejectors (n = 4). CD4+CD25^high^ and CD4+CD25^−^ control cells were isolated from patient PBMC using fluorescence activated cell sorting resulting in >95 purity. The cells were expanded using anti-CD3/CD28 coated expander beads in combination with IL-2 and IL-15 for 14–21 days. After expansion, Treg were rested for 2–3 days after which their capacity to suppress anti-donor and anti-3^rd^ party responses was evaluated in a suppression assay, in which Treg were added at increasing ratios to a newly setup MLR (with donor or 3^rd^ party stimulator cells). Proliferation was measured at day 6 using ^3^H incorporation The percentage inhibition of proliferation (y-axis) of anti-donor (left) or anti-3^rd^-party (right) responses by Treg, added at increasing ratios (x-axis) to the MLR, isolated from rejectors and non-rejectors. R vs. NR = not significant.

The suppressive capacity of peripheral blood Treg was studied in 9 patients (5 rejectors and 4 non-rejectors). CD4+CD25^high^ were isolated from PBMC. To obtain sufficient cell numbers for functional analysis, the cells were expanded using a previously validated expansion protocol [21]. Treg of each transplant patient showed potent ability to suppress both anti-donor and anti-3^rd^-party responses. Considerable variation was observed in the suppressive potential of Treg between individual patients, but overall no differences were observed between rejectors and non-rejectors (Figure 3). The expanded CD4+CD25- control T cells did not reveal suppressive capacity in any of the patients (data not shown).

### Changes in the distribution of effector and memory T cell subsets over time

**Figure 4:**
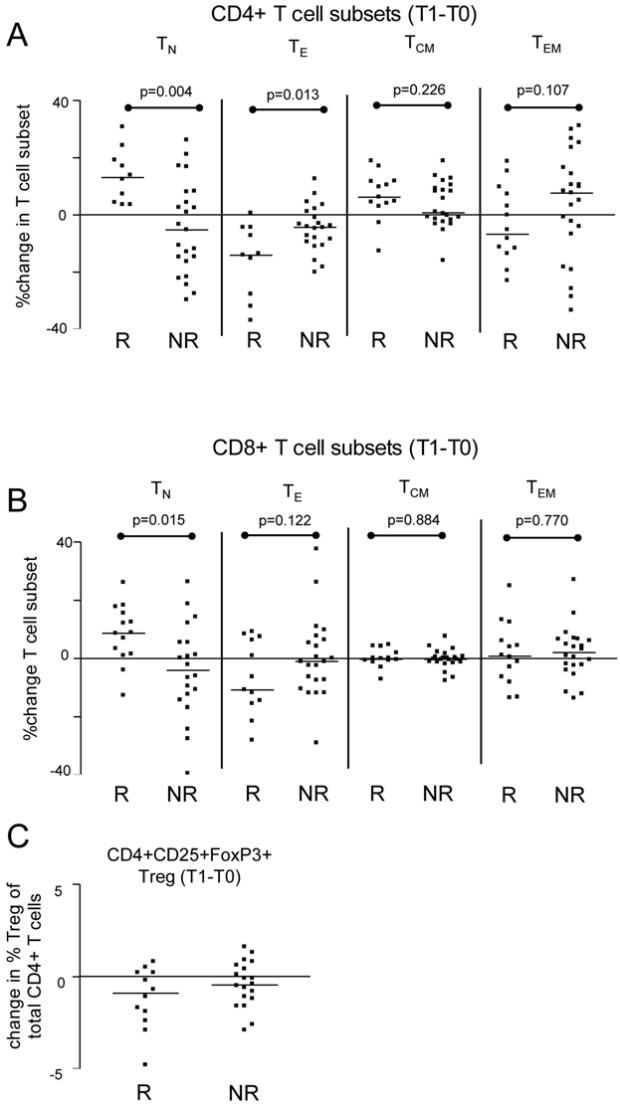
Changes in the frequency of naïve, effector, memory and regulatory T cell subsets in the peripheral blood of transplant patients between the time of transplantation (T0) and the start of tacrolimus withdrawal (T1). A positive value indicates an increase and a negative value indicates a decrease of the value in time. The change in the percentage of naïve (T_N_, CD45RA+CD62L+), effector (T_E_, CD45RA+CD62L-), central memory (T_CM_, CD45RO+CD62L+) and effector memory (T_EM_, CD45RO+CD62L-) T cell subsets within (A) the total CD4+ T cell population, and (B) the CD8+ T cell population. (C) The change in the percentage of peripheral blood CD4+CD25+FoxP3+ T cells within the total CD4+ T cell population.. R vs. NR = not significant.

Having established a difference in the ratio between memory and Treg before the reduction of tacrolimus (T1), we wondered whether changes over time would provide additional information. Analysis of the immunological markers before transplantation (T0), showed no differences between rejectors and non-rejectors. Interestingly, with regard to CD4+ T cells, all rejectors showed a relative increase between T0 and T1 in the percentage of naive T cells and all but one showed a decrease in the percentage of effector T cells, while in non-rejectors the pattern of changes was diverse (Figure 4A). In the CD8+ T cell compartment, there were comparable shifts in T cell subset percentages, but less outspoken than in the CD4+ T cell population (Figure 4B).

For both rejectors and non-rejectors, the median percentage of Treg within the CD4+ T cells was lower prior to tacrolimus reduction (1.6% and 2.0%, respectively) compared to pretransplant values (2.6% and 2.9%, respectively, p<0.01) (Figure 4C).

Taken together, a decrease in the percentage of naïve T cell levels identified transplant patients at low risk for acute rejection following reduction of immunosuppression.

### Changes in the functional capacity of CD4+ and CD8+ T cells over time

**Figure 5:**
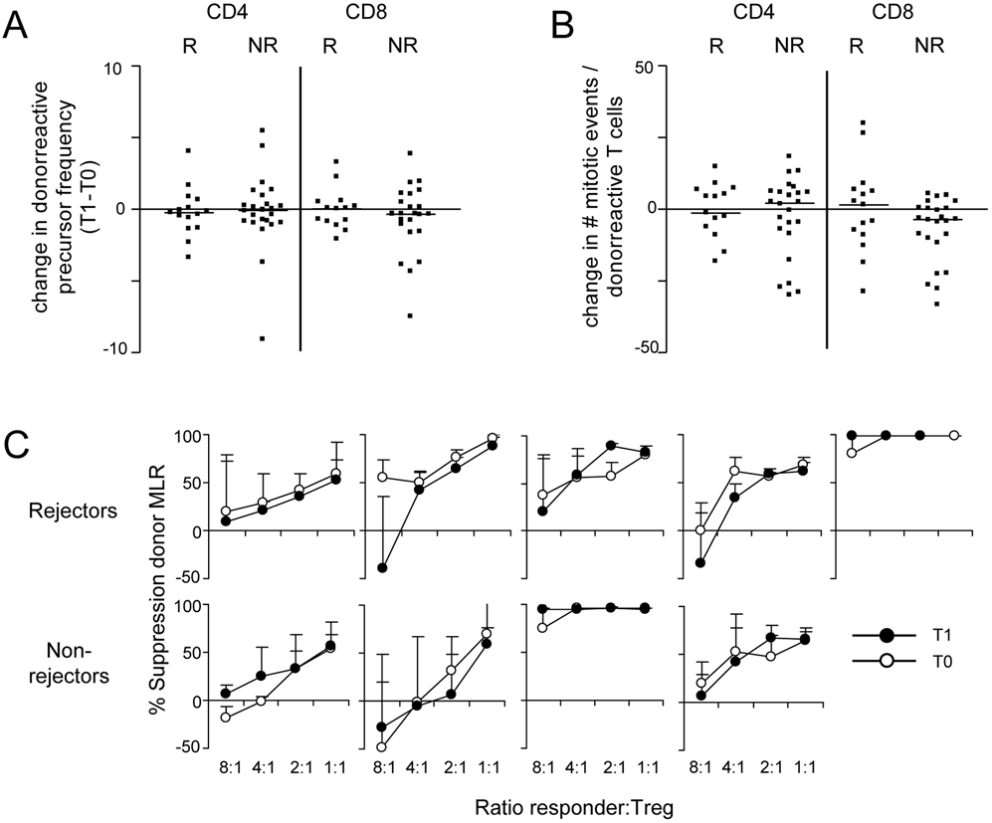
Changes in the functional capacity of T cells isolated from the peripheral blood of transplant patients between the time of transplantation (T0) and the start of tacrolimus withdrawal (T1). A positive value indicates an increase and a negative value indicates a decrease of the value in time. A CFSE MLR in combination with Modfit LT™ software was used to calculate the precursor frequency and the number of mitotic events of allo- and 3^rd^-party-reactive CD4+ and CD8+ T cells. (A) The change (y-axis) in the precursor frequency of alloreactive CD4+ and CD8+ T cells. (B) The change in the number of mitotic events (ME) of CD4+ and CD8+ alloreactive T cells. (C) The functional capacity to suppress anti-donor responses of Treg isolated before transplantation (T0, white circles) and before the start of tacrolimus reduction (T1, black circles) in 5 rejectors (upper graphs) and 4 non-rejectors (lower graphs).

The precursor frequency and mitotic events of donor and 3^rd^ party reactive CD4+ and CD8+ T cells showed little variation over time and the changes did not differ between rejectors and non-rejectors (Figures 5A and 5B). In addition, the ability of isolated Treg to suppress alloreactivity was comparable at the time of transplantation and the start of tacrolimus withdrawal (Figure 5C).

### Predictive value of immunological markers for the occurrence or freedom of rejection

As stated above, two markers were associated with successful reduction of immunosuppression: 1) a low ratio (<20) of memory CD4+ T cells: Treg prior to the start of immunosuppression reduction, and 2) a decrease in the percentage of naive T cells before start of tacrolimus reduction compared to pre-transplant values. Combination of these two markers resulted in a test with a high predictive value. We defined a positive test result (associated with rejection) as the absence of both markers, and a negative test result (associated with absence of rejection) as the presence of one or both markers. Both markers were available in 33 patients (12 rejectors and 21 non-rejectors). The sensitivity of the test for detecting a subsequent rejection was 100% (12/12) while the specificity was 76% (16/21). The positive predictive value (markers absent = positive test) with respect to subsequent rejection was 76% (12/17), while the negative predictive value (one or both markers present = negative test) was 100% (16/16). In conclusion, the combination of the two biomarkers was very useful to identify patients in whom the immunosuppressive therapy could be safely reduced.

## Discussion

In this case-control study, we have evaluated a number of T cell markers to predict the occurrence of acute renal allograft rejection following the discontinuation of tacrolimus. We did not aim to study the kinetics of T cell populations during rejection and after treatment thereof. Rather, we tried to identify markers of a state of allograft acceptance that may allow minimization of immunosuppression. In samples taken immediately before the withdrawal of tacrolimus, the ratio between memory CD8+ or memory CD4+ T cells and Treg was significantly higher in patients who experienced a rejection episode compared to non-rejectors. Apparently, an enhanced memory T cell: Treg ratio did not result in rejection as long as sufficient immunosuppression was provided. Reduction of immunosuppression tipped the balance towards rejection in a number of these patients. Our findings underscore the importance of a balance between effector/memory and regulatory mechanisms in the maintenance of immune homeostasis as has been demonstrated in several studies [27]–[29].

In addition, we observed that the change in T cell subset distribution between transplantation and the initiation of tacrolimus withdrawal was different between rejectors and non-rejectors. In the group of rejectors, an increase over time was observed in the percentage of naive T cells in the peripheral blood, with a reciprocal decrease in the percentage of effector T cells. Although more pronounced for CD4+ T cells, this phenomenon was also observed for CD8+ T cells. Without the analysis of other immune compartments and graft tissue, it is difficult to interpret these intriguing findings.

The combination of the memory T cell: Treg ratio and the changes in T cell subsets over time, resulted in a test, that is highly sensitive in the detection of patients that will develop a rejection after the withdrawal of tacrolimus at six months after renal transplantation. Accordingly, when confirmed in another study, this test could be very useful to identify renal transplant patients in whom immunosuppression can be safely reduced.

Until now, in vitro monitoring tools in solid organ transplantation have mostly been used to predict the course early after transplantation, and the most relevant information has been provided by functional assays of anti-donor reactivity [30]. One study concerned the prediction of acute rejection following reduction of immunosuppression by measuring CTLp frequencies [13]. The authors found that low CTLp frequencies (<10/10*10^6^ PBMC) identified patients in whom immunosuppression could be safely reduced. The low CTLp frequencies observed by van Besouw et al. might be viewed as a state of immune quiescence, which in some way appeared to be reflected by a low memory T cell: Treg ratio in our study. Combining the assessment of CTLp frequencies and memory T cell: Treg ratios might provide a strong monitoring tool.

Although the results of our study are based on a relatively small sample size, similar studies with comparable or larger number of patients have not been published. It is important that our findings are reproduced in a second validation cohort of patients before they can be used for the management of patients. We recognize that in a number of the assays we used, overall reactivity rather than donor reactivity was measured. In clinical practice however, monitoring tools will especially be welcomed if they are easy and fast to perform, give reproducible results, require small blood volumes of the patient, and do not require donor material.

In conclusion, in this study we have monitored a variety of phenotypic and functional T cell markers in the peripheral blood of renal transplant patients in order to find a marker that would help to predict the occurrence of acute rejection following the discontinuation of tacrolimus. Flow cytometric T cell analysis was found to be the most informative in our study and revealed an association between the occurrence of rejection and 1) the ratio of memory T cells and Treg immediately prior to the start of tacrolimus reduction and 2) changes in the distribution of naive, effector and memory T cells over time. These findings may contribute to the development of in vitro monitoring tools to identify transplant patients in whom immunosuppression can be safely reduced in order to avoid long-term side effects.
